# PPARD May Play a Protective Role for Major Depressive Disorder

**DOI:** 10.1155/2021/5518138

**Published:** 2021-04-21

**Authors:** Tao Yang, Juhua Li, Liyuan Li, Xuehua Huang, Jiajun Xu, Xia Huang, Lijuan Huang, Kamil Can Kural

**Affiliations:** ^1^Mental Health Center, West China Hospital, Sichuan University, Chengdu, China; ^2^School of Systems Biology, George Mason University, Manassas, VA 20110, USA

## Abstract

Activation of PPARD has been shown to inhibit depressive behaviors and enhances neurogenesis. However, whether PPARD is involved in the pathological development of major depressive disorder (MDD) is largely unknown. To explore the potential connection between PPARD and MDD, we first conducted a literature-based data mining to construct a PPARD-driven MDD regulating network. Then, we tested the PPARD expression changes in MDD patients from 18 independent MDD RNA expression datasets, followed by coexpression analysis, multiple linear regression analysis, and a heterogeneity analysis to study the influential factors for PPARD expression levels. Our results showed that overexpression of PPARD could inhibit inflammatory cytokine signaling pathways and the ROS and glutamate pathways that have been shown to play important roles in the pathological development of MDD. However, PPARD could also activate nitric oxide formation and ceramide synthesis, which was implicated as promoters in the pathogenesis of MDD, indicating the complexity of the relationship between PPARD and MDD. PPARG presented significant within- and between-study variations in the 18 MDD datasets (*p* value = 0.97), which were significantly associated with the population region (country) and sample source (*p* < 2.67*e* − 5). Our results suggested that PPARD could be a potential regulator rather than a biomarker in the pathological development of MDD. This study may add new insights into the understanding of the PPARD-MDD relationship.

## 1. Introduction

Major depressive disorder (MDD), also known as depression, is a mental disorder characterized by at least two weeks of pervasive low mood. The leading cause of MDD is believed to be a combination of genetic and environmental factors [1–7], with about 40% of the risk related to genetics [4].

Peroxisome proliferator-activated receptor beta/delta (PPARD) is one of the three known PPARs (the others are PPAR*α* and PPAR*γ*), which are part of the nuclear receptor superfamily of transcription factors. PPARD governs diverse biological processes [8] and shows a widespread brain expression, with particularly high levels in the hippocampus, entorhinal cortex, and hypothalamus [9, 10].

Several previous studies show that PPARD might be involved in depression occurrences [11, 12]. Specifically, the hippocampal genetic knockdown of PPARD has been shown to cause depression-like behaviors and neurogenesis suppression [12], suggesting that PPARD plays a crucial role in neurogenesis and regulates both depression and memory. Moreover, hippocampal PPARD overexpression or activation inhibits stress-induced depressive behaviors and enhances neurogenesis [11]. However, so far, whether PPARD is involved in MDD and its related underlying mechanism is largely unknown.

Here, we hypothesized that PPARD could play a role in the pathological development of MDD. Our results supported this hypothesis and indicated that deficiency of PPARD might be involved in the pathogenesis of MDD by regulating cytokine-related signaling pathways. However, our results also demonstrate the variation of PPARD expressions in the cases of MDD, which may be influenced by multiple factors, including sample postulation regions. Our study might add new insights into the understanding of the roles that PPARD plays in MDD.

## 2. Method

The rest of this study is organized as follows. First, we conducted a systematic literature-based network analysis to explore the possible relationship between PPARD and MDD. Then, we analyzed the expression of PPARD in 18 public human RNA array-expression datasets linked with MDD diagnosis. After that, we employed multiple linear regression analysis to study the potential, influential factors of PPARD expression in the cases of MDD. To facilitate the understanding of the results presented, we provided additional supporting data and information in a supplementary material named PPARD_MDD.

### 2.1. Literature-Based Pathway Analysis

Assisted by Pathway Studio (PS) (http://www.pathwaystudio.com; version 12.3.0.16), we conducted a systematical pathway analysis to uncover PPARD-driven MDD regulators at different levels, including proteins, small molecules, complexes, and functional classes. Owned by Elsevier Inc., the PS database ResNet [13] contains functional relationships and pathways of mammalian proteins, including human, mouse, and rat genes. The database covers over 24 million PubMed abstracts and 3.5 million Elsevier and 3rd part full-text papers.

The MDD regulators were identified by using the Shortest Path Module within Pathway Studio (https://supportcontent.elsevier.com/Support%20Hub/Pathway%20Studio/Guide%20to%20Building%20Pathways%20in%20Mammal%20with%20Pathway%20Studio%20Web.pdf). Each relation between MDD and its regulators was supported by one or more references, as shown in Ref4Pathway in the Supplementary Materials (available here). The sentences from each supporting reference were manually checked for quality control. Following the same process, the items influenced by PPARD were also identified, with the overlapped items used to build the PPARD-driven MDD regulating network.

The following criteria were applied for the selection of the PPARD-driven MDD regulators. (1) The direction is from PPARD to MDD. (2) Each relationship (network edge) has a signed polarity (positive or negative effect). (3) The quality control of each relationship (network edge) was conducted through manual inspection of the supporting references. (4) The type of regulators includes genes (proteins), functional class, and small molecules. For a relationship with more than 10 supporting references, we inspected the first 10 references. The relationships that passed the filtering criteria were employed to construct the PPARD-driven signaling pathways that may affect roles in the pathology of MDD. We provided the details of these identified relationships and the underlying supporting references in Ref4Pathway in the Supplementary Materials, including the reference title and the sentences where a relationship has been identified.

### 2.2. MDD RNA Expression Data Acquisition

To explore the quantitative change of PPARD in MDD patients and test whether PPARD could work as a biomarker for MDD, we conducted an MDD RNA expression data-based analysis on PPARD expression. We acquired MDD RNA array-expression datasets from GEO (https://www.ncbi.nlm.nih.gov/geo/). Initially, we searched with the keyword “major depressive disorder” and identified 317 studies with series data. Then, the following criteria were applied to fulfill the purpose of this study, including the following:

1. The data type was RNA expression by array
2. The organism of datasets was *Homo sapiens*
3. The study design was MDD vs. healthy control
4. The total number of samples was not less than 10
5. The dataset and corresponding format files were feasibly available and downloadable

There were 18 datasets that satisfied the selection criteria and were included for expression analysis. We provided the information employed in this study of these datasets in Table 1, and the GEOID can be used to retrieve the detailed description of each dataset at https://www.ncbi.nlm.nih.gov/geo/.

### 2.3. Expression of PPARD in MDD RNA Expression Datasets

In this study, the expression for PPARD was estimated for each of the 18 datasets listed in Table 1. Specifically, we first calculated the fold change that was defined as the ratio between the mean expression of MDD cases and that of healthy controls. Then, the log2-transferred fold change (LFC) was used as effect size, such that fold changes lower than one become negative, while those greater than one become positive. The significance criteria were set as abs (LFC) ≥ 1 and *p* < 0.05.

### 2.4. Coexpression Analysis

Using the 18 MDD RNA expression datasets, we also studied the coexpression between PPARD and its driven genes regulating MDD. The purpose of the coexpression analysis was to validate the relationships between PPARD and its driven genes at the gene expression level. In the datasets where PPARD showed a small effect size (LFC ∈ [−0.3,0.3]), we assumed that PPARD exerted no influence on its driven genes. Thus, the analysis only focused on PPARD with significant changes.

### 2.5. Heterogeneity Analysis of PPARD Expression

A heterogeneity analysis was conducted to study the variance within and between different studies [14] to determine if there was a significant between-study variance compared with within-study variance. The analysis was conducted by using MATLAB (R2017a) with the results presented in ExpressionOfPPARD in the Supplementary Materials.

### 2.6. Multiple Linear Regression Analysis

To investigate the possible influential factors for the gene expression of PPARD in the case of MDD, we conducted a multiple linear regression (MLR) analysis on five parameters, including sample size, sample population region (country), sample source, data acquisition platform, and study age. *p* value < 0.05 was set as a significance criterion for the identification of significant factors. The analysis was performed using the statistic toolbox “regress()” in MATLAB (R2017a).

## 3. Results

### 3.1. PPARD-Driven Network

**Figure 1:**
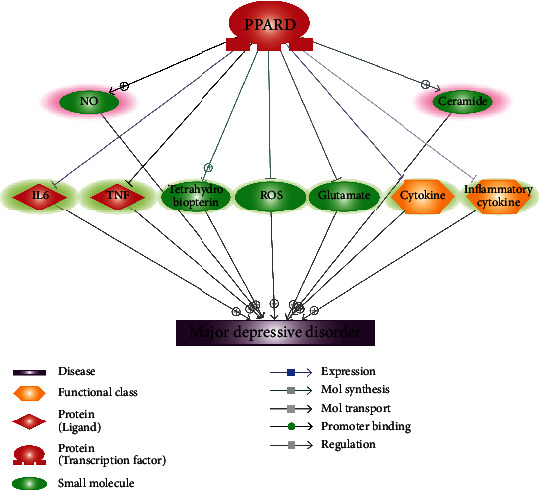
PPARD-driven pathways involved in the pathology of MDD. The pathway was built through Pathway Studio-assisted literature data mining, supported by over 400 references. The items highlighted in green are the ones that were driven by PPARD to suppress the development of MDD, and the red ones were regulated by PPARD to promote MDD development.

Literature-based network analysis revealed nine entities regulated by PPARD that were also upstream regulators of MDD, as shown in Figure 1. Among these entities, increased PPARD could exert a major positive influence on MDD by upregulation of one MDD inhibitors (tetrahydrobiopterin) and downregulation of 6 MDD promoters, including two cytokine genes (IL6 and TNF), two small molecules (ROS and glutamate), and the two functional classes (cytokine and inflammatory cytokine). These MDD regulators were highlighted in green in Figure 1. However, PPARD may also activate nitric oxide production (NO) and ceramide, two promoters of MDD (highlighted in red in Figure 1). Overall, these literature data mining-based relationships suggested that the deficiency of PPARD might facilitate the development of MDD by activating cytokine classes and promoting the secretion of reactive oxygen species (ROS) and glutamate. The pathways presented in Figure 1 were based on over 300 independent studies. The reference information was provided in Ref4Pathway in the Supplementary Materials. To note, over 400 references were listed as some references support multiple relationships.

Specifically, there were about 250 studies (references) supporting the PPARD → cytokine genes → MDD pathways. In vitro cell line expression studies of both human and animal models showed that PPARD reduces the expression and secretion of cytokines, including inflammatory cytokines and proinflammatory cytokines. While clinical studies and animal models showed that cytokines could induce sickness behavior with depression-like symptoms, contribute to cognitive decline, and induce MDD. Therefore, inhibition of cytokines by PPARD supports the suppression role of PPARD in the pathological development of MDD.

Moreover, there were 54 references that support the PPARD → ROS → MDD pathway. In vitro human cell line studies showed that activation of PPARD reduces radiation and angiotensin II-induced ROS generation by modulating the expression of SIRT1. And ROS have been suggested to play an important role in the pathogenesis of MDD in clinical studies and animal models.

In addition, 94 references support the PPARD → glutamate → MDD pathway. Both in vitro and human studies show that heightened glutamate plays an important role in the pathophysiology of MDD. In vitro cell line and animal studies showed that activation of PPARD inhibits glutamate release.

There were also two studies that suggested a PPARD → tetrahydrobiopterin → MDD pathway. Tetrahydrobiopterin has been reported to improve clinical depression by increasing TH activity. Activation of PPARD enhances the regenerative capacity of human endothelial progenitor cells by stimulating the biosynthesis of tetrahydrobiopterin.

On the other hand, there were 22 studies (references) that support the PPARD → NO → MDD relationship, and nine studies (references) support the PPARD → ceramide → MDD relationship. These studies suggested that increased PPARD could increase the production of NO and ceramide in the plasma of the human body. In clinical studies and animal model studies, increased production of NO and ceramide has been shown to promote the development of neuroinflammation-associated disorders, including MDD. Therefore, increased PPARD may have the promotion effect on MDD through the activation of NO and ceramide.

### 3.2. Expression Variation of PPARD in 18 MDD Expression Datasets

**Figure 2:**
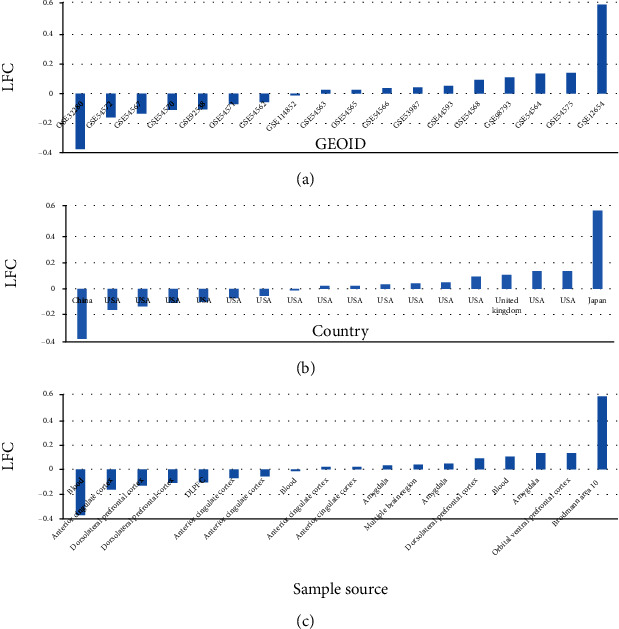
Expression of PPARD in 18 MDD RNA expression datasets: (a) the expression by datasets; (b) the expression by country; (c) the expression by sample source.

To explore the expression changes of PPARD in the cases of MDD, we calculated the LFC of PPARD in the MDD patients compared to healthy controls using 18 different RNA expression datasets, as shown in Figure 2(a). The expression of PPARD demonstrated varies among different studies, ranging from -0.38 to 0.61 (LFC = 0.013 ± 0.19). Among these datasets, seven presented mild decreased expression (LFC = −0.13 ± 0.11). The majority of datasets (10 out of 18) showed increased expression of PPARD in MDD patients compared to healthy controls (LFC = 0.13 ± 0.17). However, none of these changes was identified as significant. The datasets were collected from four different countries, eight different sample sources, and six different platforms, which may well represent different cases of MDD. Our results suggested that PPARD might not present significant changes among MDD patients. For more details of the PPAR expression data analysis, please refer to ExpressionOfPPARD in the Supplementary Materials.

### 3.3. Coexpression Analysis

For the datasets that showed the lowest PPARD expression levels (GSE32280: LFC = −0.38) and highest expression levels (GSE12654: LFC = 0.60), PPARD demonstrated a significant negative correlation with IL6 (Fisher *Z* transferred Pearson *r* = −0.41; *p* = 0.030) and TNF (Fisher *Z* transferred Pearson *r* = −0.38; *p* = 0.035). These results indicated that when PPARD expression got activated, TNF expression was inhibited, helping the suppression of MDD. On the other hand, when PPARD was downregulated, IL6 presented overexpression, promoting the development of MDD. We assumed that, when PPARD showed small expression changes (LFC ∈ (−0.3,0.3)), it had limited influence on either TNF or IL6. Thus, coexpression analysis was not effective in evaluating the relation between PPARD and these two genes. We provided the results in ExpressionOfPPARD in the Supplementary Materials. Our results support the PPARD → TNF and PPARD ➔ IL6 regulation identified in Figure 1.

### 3.4. Multiple Linear Regression Analysis Results

**Figure 3:**
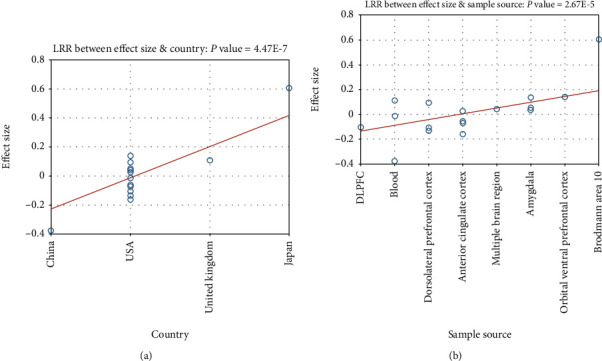
Multiple linear regression analysis results for the influential factors of PPARD expression in the cases of major depressive disorder: (a) results for population regions (country); (b) results for sample source.

MLR results showed that out of the five factors tested, only the population region (country) and sample source were significant influential factors (*p* = 4.47*E* − 07 and 2.67*E* − 05, respectively) for PPARD expression in MDD patients, as shown in Figure 3. However, the other three factors, namely, sample size, study age, and platform, were not significant factors for the expression of PPARD in the case of MDD (*p* > 0.15). For the details of MLR results, please refer to MLR_Results in the Supplementary Materials.

### 3.5. Heterogeneity Analysis

**Figure 4:**
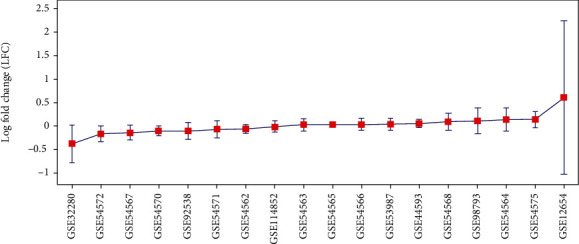
Error bar plot of the within-study variance of the PPARD expression among 18 major dispersive disorder RNA expression datasets.

The heterogeneity analysis was employed to test whether the total variance mainly resulted from between-study variance or from both within- and between-study variance. The analysis results showed that the total variance among different studies was 7.9, which was smaller than the expected variance (17) given that all studies have the same actual effect. Our results indicated that the between-study variance was not the primary source contributing to total variance among these studies (*p* value = 0.96). In other words, there were significant within-study variances among these datasets, as shown in Figure 4. To note, the dataset GSE12654 that presented the highest averaged expression levels (LFC = 0.60) also demonstrated the most significant within-study variance (STD = 1.63). For more details of these results, please refer to ExpressionOfPPARD in the Supplementary Materials. Our results indicated that the variation might partially cause the overall nonobvious expression changes of PPARD in MDD patients among samples within each study.

## 4. Discussion

In this study, we explored the possible relationship between PPARD and MDD through literature-based network analysis and RNA expression variation analysis of PPARD in the cases of MDD. Moreover, we employed multiple linear regression analysis and heterogeneity analysis to study the potential, influential factors of PPARD expression in the cases of MDD. Coexpression analysis between PPARD and its driven genes was conducted to provide partial validation of the PPARD-driven MDD regulating pathway. The literature-based pathway in Figure 1 supports the hypothesis that PPARD might be involved in the pathogenesis of MDD by regulating cytokine-related signaling pathways. However, our results also demonstrated the variation of PPARD expressions in the cases of MDD, which may be influenced by multiple factors, including sample postulation regions and sample sources. These results suggested that PPARD might be a regulator rather than a biomarker for the pathological development of MDD.

Firstly, literature-based network analysis showed that PPARD might influence multiple molecules that functionally regulate MDD, mostly in a beneficial way (Figure 1). Our results were consistent with previous studies that PPARG plays a crucial role in regulating depression and depressive behaviors [11, 12]. Most noticeably, PPARG was shown to inhibit multiple cytokine signaling pathways, which have been demonstrated to play an important role in the pathophysiology of MDD [15]. On the one hand, PPARD activation blocks the synthesis of inflammatory cytokines, including IL1*β*, IL6, and TNF*α* [16], which explains the fact that PPARD agonists downregulate the expression of these cytokines [17]. On the other hand, serum TNF*α*, IL6, and IL1*β* were implicated as important factors in the psychopathology of acute-phase MDD [18], which were found to stimulate behavioral changes of MDD [19]. These findings support the PPARD-cytokine signaling-MDD pathways, where increased expression of PPARD plays an inhibitive role in MDD.

Moreover, the pathway analysis also revealed that PPARD inhibits two small molecules that were the promoters of MDD, namely, free oxygen radicals (ROS) and glutamate (Figure 1). Activation of PPARD was found to counteract angiotensin II-induced ROS generation and modulates glutamate release [20–22], which have been suggested to play essential roles in the pathophysiology of MDD [23, 24]. PPARD activation also stimulates the biosynthesis of tetrahydrobiopterin [25], which was implicated to play a role in clinical depression [26]. These findings suggested additional pathways where PPARD plays beneficial functions in the pathological development of MDD.

However, our pathway analysis also revealed that PPARD activation promotes the nitric oxide (NO) formation and ceramide synthesis [27, 28], which were found to play important roles in the neurobiology of major depression [29, 30]. These findings suggested the complicity of the relationship between PPPARD and MDD.

Coexpression analysis suggested that decreased expression of PPARD in MDD patients might lead to elevated IL6 expression, while overexpression of PPARD could suppress the expression of TNF. Both IL6 and TNF encode cytokines that have been shown to play a key role in the pathogenesis of MDD [31]. These findings support a potential PPARD → cytokine → MDD signaling pathway that has been identified through literature data mining (Figure 1).

Expression data analysis showed that PPARD only demonstrated mild variations among 18 different MDD datasets (LFC = −0.38 to 0.61), with 55.56% of studies presenting overexpression and 44.44% studies showing reduced expression. As the datasets were collected from four different countries and eight different sample sources and using six different platforms, our study results may well represent different cases of MDD. Our results suggested that PPARD might not be a biomarker for the pathological development of MDD. Although the deficiency of PPARD might lead to depression-like behaviors and promote the development of MDD, it may not naturally happen in the majority of MDD patients. We presented the details of the PPAR expression in ExpressionOfPPARD in the Supplementary Materials.

MLR analysis showed that the population region (country) and sample source were significant, influential factors (*p* = 4.47*E* − 07 and 2.67*E* − 05, respectively) of PPARD expression levels in the case of MDD. Moreover, a heterogeneity analysis indicated that significant within-study variance might exist among individual MDD patients (see Figure 4), which is worthy of further study. However, due to the lack of clinical information of the 18 expression datasets, the related analysis was not conducted in this study.

This study has several limitations that need further investigation. First, the pathways built (Figure 1) were based on previous studies. Although coexpression analysis provided partial validation of the pathway, biology experiments are needed to test the relationships identified. Second, more clinical parameters (e.g., age, gender, disease stage, and drug status) should be tested regarding their influence on MDD expression variation.

## 5. Conclusion

This study was among the first studies to explore the relationship between PPARD and MDD. The literature-based pathway built here supported a potential PPARD → MDD relationship that is worthy of further investigation. However, PPARD might not be a biomarker for MDD at the gene expression level.

## Figures and Tables

**Table 1:** The 18 major depression disorder RNA expression datasets from GEO.

Dataset GEOID	Data contributors	# control/cases	Country	Study age	Platform	Sample source	Sample organism
GSE12654	Iwamoto et al., 2008	15/11	Japan	13	GPL8300	Brodmann area	*Homo sapiens*
GSE32280	Yi et al., 2012	8/16	China	9	GPL570	Blood	*Homo sapiens*
GSE44593	Sibille et al., 2016	14/14	USA	5	GPL570	Amygdala	*Homo sapiens*
GSE53987	Lanz et al., 2014	18/16	USA	7	GPL570	Multiple brain region	*Homo sapiens*
GSE54562	Sibille et al., 2014	10/10	USA	7	GPL6947	Anterior cingulate cortex	*Homo sapiens*
GSE54563	Sibille et al., 2014	25/25	USA	7	GPL6947	Anterior cingulate cortex	*Homo sapiens*
GSE54564	Sibille et al., 2014	21/21	USA	7	GPL6947	Amygdala	*Homo sapiens*
GSE54565	Sibille et al., 2014	16/16	USA	7	GPL570	Anterior cingulate cortex	*Homo sapiens*
GSE54566	Sibille et al., 2014	14/14	USA	7	GPL570	Amygdala	*Homo sapiens*
GSE54567	Sibille et al., 2014	14/14	USA	7	GPL570	Dorsolateral prefrontal cortex	*Homo sapiens*
GSE54568	Sibille et al., 2014	15/15	USA	7	GPL570	Dorsolateral prefrontal cortex	*Homo sapiens*
GSE54570	Sibille et al., 2014	13/13	USA	7	GPL96	Dorsolateral prefrontal cortex	*Homo sapiens*
GSE54571	Sibille et al., 2014	13/13	USA	7	GPL570	Anterior cingulate cortex	*Homo sapiens*
GSE54572	Sibille et al., 2014	12/12	USA	7	GPL570	Anterior cingulate cortex	*Homo sapiens*
GSE54575	Sibille et al., 2014	12/12	USA	7	GPL96	Orbital ventral prefrontal cortex	*Homo sapiens*
GSE92538	Hagenauer et al., 2016	56/29	USA	5	GPL10526	DLPFC	*Homo sapiens*
GSE98793	Kelly et al., 2017	64/128	UK	4	GPL570	Blood	*Homo sapiens*
GSE114852	Breen et al., 2018	85/31	USA	3	GPL10558	Blood	*Homo sapiens*

Note: “study age” of a dataset was defined as the current year—the year of data submission.

## Data Availability

The data used or analyzed during the current study are available from the corresponding author on a reasonable request.

## Supplementary Materials

Supplementary Materials The supplementary materials is a multiworksheet Excel file that contains additional results described as follows. (1) Ref4Pathway: reference information for the network built in Figure 1. (2) ExpressionOfPPARD: the expression of PPARD in 18 MDD RNA expression datasets and the coexpression analysis between PPARD and IL6 and TNF. (3) MLR_Results: the multiple linear regression analysis results for the potential factors influencing the PPARD expression in MDD patients. The Excel file is available online at http://www.gousinfo.com/database/Data_Genetic/PPARD_MDD.xls.
