# Supplementary material for: PPARD May Play a Protective Role for Major Depressive Disorder
Source: PPAR Res. 2021 Apr 21;2021:5518138. doi: 10.1155/2021/5518138 (PMC8081621; doi:10.1155/2021/5518138)
Supplement: Supplementary Materials — The supplementary materials is a multiworksheet Excel file that contains additional results described as follows. (1) Ref4Pathway: reference information for the network built in Figure 1. (2) ExpressionOfPPARD: the expression of PPARD in 18 MDD RNA expression datasets and the coexpression analysis between PPARD and IL6 and TNF. (3) MLR_Results: the multiple linear regression analysis results for the potential factors influencing the PPARD expression in MDD patients. The Excel file is available online at http://www.gousinfo.com/database/Data_Genetic/PPARD_MDD.xls. [file 5518138.f1.pdf]

Supplementary Material: PPARD_MDD

The Supplementary Materials is a multi-worksheet excel file that contains additional results described as follows.

1) Ref4Pathway: Reference information for the network built in Figure 1.
2) ExpressionOfPPARD: The expression of PPARD in 18 MDD RNA expression datasets and the co-expression analysis between PPARD and IL6 and TNF.
3) MLR results: The multiple linear regression analysis results for the potential factors influencing the PPARD expression in MDD patients.

The excel file is online available at: www.gousinfo.com/database/Data_Genetic/PPARD_MDD.xlsx
